# Biomechanical Evaluation and Comparative Analysis of Functional Scores in Suture Anchor Versus Transosseous Tunnel Patellar Tendon Repair Techniques in Adult Patients: A Systematic Review and Meta-Analysis

**DOI:** 10.7759/cureus.73495

**Published:** 2024-11-11

**Authors:** Ahmed Elnewishy, Abdelfatah M Elsenosy, Hagar Teama, Mohamed Salem

**Affiliations:** 1 Orthopaedics, Royal Berkshire Hospital NHS, Reading, GBR; 2 Orthopaedic Surgery, Elkasr Elainy Medical School, Kafr Elsheikh, EGY; 3 Trauma and Orthopaedics, University Hospitals Dorset NHS Foundation Trust, Poole, GBR; 4 Pharmacy, Kafr Elsheikh Hospital, Kafr Elsheikh, EGY; 5 General Surgery, King's Mill Hospital, Nottingham, GBR

**Keywords:** biomechanical properties, functional outcomes, patellar tendon repair, suture anchor, transosseous tunnel

## Abstract

Patellar tendon injuries, although less frequent than other knee injuries, can have a profound impact on knee function, often leading to significant disability. Among the various surgical techniques employed to repair these injuries, the suture anchor and transosseous tunnel methods are the most commonly used. This systematic review and meta-analysis compares the biomechanical properties and functional outcomes of these two repair techniques. A thorough search of relevant databases was conducted until June 2024, including studies that directly compared the suture anchor and transosseous tunnel methods for patellar tendon repair. Data extraction and quality assessments were performed independently by two reviewers. The primary outcomes assessed were biomechanical strength and functional recovery, using the International Knee Documentation Committee (IKDC) scores, Lysholm score, and Kujala score. A total of four studies involving 196 participants met the inclusion criteria. Both techniques were found to significantly improve functional outcomes. However, suture anchor repairs were associated with lower complication rates and earlier rehabilitation compared to the transosseous tunnel technique. Despite these differences, the meta-analysis showed no statistically significant difference in postoperative IKDC scores between the two methods. In conclusion, while both the suture anchor and transosseous tunnel techniques are effective for patellar tendon repair, the suture anchor method appears to offer biomechanical advantages, fewer complications, and faster recovery. Further high-quality research is recommended to validate these findings.

## Introduction and background

Patellar tendon injuries, while less frequent than other knee extensor mechanism injuries, can result in substantial functional impairments and a prolonged recovery period. These injuries are particularly prevalent among younger, active individuals, especially males involved in high-impact sports such as basketball and soccer. Beyond functional limitations, patellar tendon injuries impact patient-centered outcomes, including quality of life and overall patient satisfaction with recovery. Effective rehabilitation protocols, particularly physiotherapy, play a crucial role in promoting recovery and preventing complications. Early and structured postoperative rehabilitation helps enhance joint stability and functional outcomes, directly influencing patients' quality of life and long-term satisfaction with surgical interventions. Therefore, a comprehensive approach to evaluating repair techniques must consider these patient-centered outcomes and the influence of postoperative care in addition to biomechanical stability.

The patellar tendon plays a crucial role in knee extension, which is necessary for movements such as jumping and rapid changes in direction [1]. Acute ruptures of the patellar tendon typically occur following a sudden, forceful contraction of the quadriceps muscle during athletic activity. In contrast, chronic injuries usually result from repetitive microtrauma that progressively weakens the tendon over time [2]. While patellar tendon injuries are less frequent than Achilles tendon injuries, they account for approximately 5% of all knee extensor mechanism injuries, underscoring their importance in knee mechanics [3].

Recent trends suggest an increasing incidence of patellar tendon ruptures, likely driven by a combination of an aging yet still active population and advances in diagnostic practices. Between 2001 and 2020, there was a notable rise in patellar tendon injuries, particularly among older adults [4]. Diagnosis is primarily based on clinical examination, although imaging studies, such as magnetic resonance imaging (MRI) or ultrasound, are commonly employed to confirm the severity of the injury. Key symptoms typically include pain, swelling, and the inability to fully extend the knee or perform a straight leg raise [5].

In recent years, the suture anchor technique has gained increasing popularity for repairing the patellar tendon. This method involves securing high-strength sutures into the bone to reattach the tendon, a technique that has been shown to reduce gap formation during cyclic loading and improve the tendon's ultimate load-bearing capacity when compared to traditional transosseous tunnel repairs [6]. Suture anchor repairs are also associated with lower rerupture rates, which further supports their growing use in tendon repair [7].

The transosseous tunnel technique, however, has long been regarded as the gold standard for patellar tendon repair. It involves creating bone tunnels through which sutures are passed to fix the tendon. Despite its widespread use, recent research suggests that this technique may result in larger gaps and a lower ultimate load-to-failure result than suture anchor techniques [8]. Nevertheless, the transosseous tunnel method remains a valuable option, especially in cases where tissue quality is compromised or the use of suture anchors is not recommended [9].

In more complex or chronic cases, tendon repair often requires reinforcement through augmentation techniques. These may involve the use of autografts, allografts, or synthetic materials to strengthen the repair. For example, the combination of an ipsilateral semitendinosus autograft with suture tape augmentation has been shown to improve the repair strength of the healing tendon [10]. In a study by Rothfeld et al. [11], the use of knotless suture anchor internal braces to reinforce the repair was found to offer biomechanical advantages compared to traditional repair methods.

Comparative studies have demonstrated that suture anchor repairs result in less gap formation during cyclic loading and comparable ultimate load-to-failure when evaluated against transosseous tunnel repairs. Additionally, systematic reviews and meta-analyses have reported lower cyclic gap displacement with suture anchor fixation, although no significant difference in ultimate load-to-failure has been observed between the two techniques [6]. Another biomechanical study concluded that while both techniques achieved similar load-to-failure results, suture anchor repairs led to less gap formation, potentially contributing to improved initial stability [9].

Clinically, one of the key advantages of suture anchor repairs over transosseous tunnel repairs is the lower rate of reruptures. Large cohort studies have shown rerupture rates of 0% for suture anchor repairs compared to 7.5% for transosseous tunnel repairs [7]. Furthermore, patient-reported outcomes, such as subjective functional assessments and International Knee Documentation Committee (IKDC) scores, consistently favor suture anchor repairs. For instance, in studies comparing quadriceps tendon repairs using suture anchors versus transosseous tunnels, the suture anchor group reported higher subjective function and improved IKDC scores [12].

## Review

Objective

The objective of this systematic review and meta-analysis is to comprehensively compare the biomechanical, functional, and patient-centered outcomes of suture anchor versus transosseous tunnel techniques in patellar tendon repair. This review evaluates the quality of life, patient satisfaction, and functional recovery to provide a well-rounded comparison of both methods. Additionally, the study considers the role of postoperative rehabilitation protocols, such as physiotherapy, which are critical to patient recovery and satisfaction. By examining these elements, this analysis aims to guide clinical decision-making for optimal patient outcomes.

Methods

Search Strategy

In June 2024, we conducted a comprehensive search across multiple databases, including PubMed, Scopus, Google Scholar, and the Cochrane Library, to identify studies relevant to patellar tendon repair techniques. To minimize publication bias and increase the comprehensiveness of the review, we expanded our search to include grey literature sources, such as conference abstracts, theses, and relevant non-commercial reports from sources such as OpenGrey and ProQuest Dissertations & Theses Global. We specifically targeted studies comparing suture anchor and transosseous tunnel techniques. Reference lists from selected studies were also reviewed for additional sources. The search terms combined MeSH and free-text keywords such as "patellar tendon repair", "suture anchor", "transosseous tunnel", "biomechanical performance", and "functional outcomes". Boolean operators (AND, OR) were used to refine the results, and filters limited the search to English-language studies published in the last 10 years.

Inclusion Criteria

We included any peer-reviewed clinical study that directly compared the suture anchor and transosseous tunnel techniques for patellar tendon repair. The selected studies included randomized controlled trials, cohort studies, case studies, and observational studies. The inclusion criteria aimed to capture a broad spectrum of patient demographics and clinical settings, ensuring a thorough and diverse analysis of treatment outcomes.

Exclusion Criteria

Studies were excluded if they were not peer-reviewed, such as reviews, editorials, or opinion pieces, or if they did not directly compare the two surgical techniques. Additionally, studies published in languages other than English were excluded to maintain uniformity across the review process.

Outcome Measures

The primary outcomes evaluated in this review focused on both functional and patient-centered recovery indicators. Functional recovery was assessed through standardized scoring systems such as the IKDC score, Lysholm score, Tegner activity score, and the knee injury and osteoarthritis outcome score (KOOS), as well as pain levels measured via the visual analog scale (VAS). These functional scores were chosen due to their extensive validation and widespread use in assessing knee stability, pain, and overall function post-tendon repair, providing robust insights into biomechanical efficacy.

To capture patient-centered experiences, we also evaluated outcomes related to quality of life and patient satisfaction, as these provide insight into the personal impact of each repair technique and inform long-term recovery expectations. Secondary outcomes included rerupture rates, complication rates, and reoperation rates, which contribute to understanding the safety profile of each technique. These measures were selected to give a comprehensive overview of both clinical and patient-centered outcomes, thus supporting a holistic understanding of each technique's effectiveness.

Data Extraction and Quality Assessment

Two reviewers independently extracted data from the included studies using a predefined extraction form, capturing details such as author information, year of publication, study design, sample size, patient demographics, intervention details, outcomes, complications, and follow-up duration.

The quality of the studies was assessed using the Newcastle-Ottawa Scale (NOS) for non-randomized studies, which evaluates three key aspects: selection, comparability, and outcome/exposure. Each study received a score out of a possible nine stars. To ensure a consistent standard of evidence, studies with a score of less than six were excluded from the meta-analysis, as they were deemed to have a higher risk of bias. Studies scoring between six and nine were included, with scores influencing the weight given to their findings in the analysis. This approach ensured that only studies of moderate to high quality contributed to the final analysis, providing transparency in quality appraisal and enhancing the reliability of our findings.

Statistical Analysis

The extracted data were analyzed using RevMan version 5.4 software (The Cochrane Collaboration, London, England, UK). Continuous outcomes were reported as standard mean differences (SMD) with 95% confidence intervals (CI), while categorical outcomes were presented as risk ratios (RR) with 95% CIs. The I^2^ statistic was employed to assess heterogeneity between studies. A value greater than 50% indicated significant heterogeneity, prompting the use of a random-effects model. If I^2^ was below 50%, a fixed-effects model was used [13,14]. Publication bias was assessed using funnel plots and Egger's test. A two-sided p-value of less than 0.05 was considered statistically significant [15].

Results

Search and Study Selection

Our comprehensive search process began by identifying 150 records from selected databases and grey literature sources, such as conference abstracts, theses, and relevant non-commercial reports (e.g., OpenGrey and ProQuest Dissertations & Theses Global). After removing duplicates, 130 records remained, which were screened based on titles and abstracts. At this stage, 100 records were excluded as they did not meet the specific criteria established for comparing suture anchor and transosseous tunnel techniques in patellar tendon repair.

**Figure 1:**
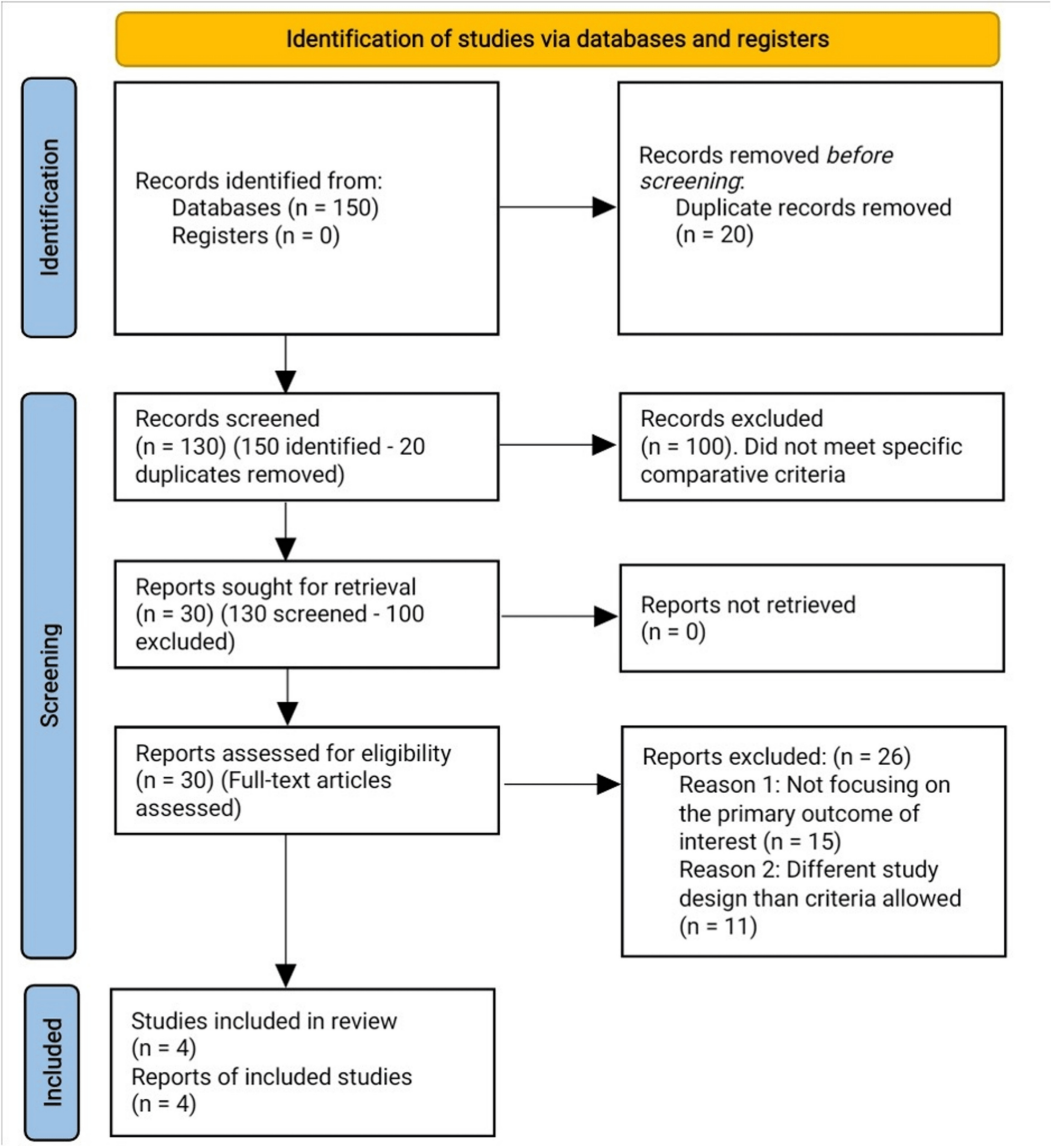
PRISMA flowchart of the reviewed studies PRISMA: Preferred Reporting Items for Systematic Reviews and Meta-Analyses

The remaining 30 articles, including both peer-reviewed publications and grey literature, were subjected to a full-text review. During this phase, 26 articles were excluded for various reasons, including lack of direct comparison between the techniques, insufficient data on relevant outcome measures, or being published in languages other than English. Additionally, studies that explored alternative approaches to patellar tendon repair or utilized weaker methodological designs were excluded. Ultimately, four studies met all the inclusion criteria and were included in the quantitative synthesis for our meta-analysis (Figure 1).

Study Characteristics

This analysis includes data from four studies involving a total of 196 participants, all of whom underwent surgical repair for patellar tendon injuries. The studies consist of two cohort studies: one prospective non-randomized controlled trial and one retrospective cohort study. Each study provided comprehensive details on patient demographics, specific interventions, follow-up durations, and outcome measures. Additionally, information on postoperative rehabilitation protocols was noted, where available, to account for variations in recovery regimens that could influence outcomes. Most studies implemented structured physiotherapy protocols that included gradual weight-bearing exercises, range-of-motion training, and strength-building activities. These protocols typically began within the first two to six weeks post-surgery, progressing based on individual recovery markers, which may have impacted the rate and extent of functional recovery.

The primary outcomes evaluated across these studies included functional scores such as the IKDC subjective scores, Lysholm scores, Tegner activity scores, Kujala scores, and KOOS. Additional assessments included congruence angles, patellar tilt angles, pain levels measured by the VAS, and isokinetic strength testing results. To enhance consistency in outcome comparisons, we focused on studies that provided detailed reporting on these scores, although slight variations existed across studies in the tools used for pain and strength assessments.

The differences in baseline patient characteristics, such as age, physical activity levels, and severity of initial tendon injury, were also considered, as these factors may contribute to variability in outcomes. For instance, two studies reported higher activity levels among participants, suggesting greater baseline knee stability, which could impact postoperative functional scores. Overall, both transosseous sutures and suture anchors yielded favorable clinical outcomes, with suture anchors showing certain advantages, particularly in facilitating earlier rehabilitation, possibly due to reduced initial gap formation and higher load-bearing capacity (see Table 1 for detailed comparisons).

**Table 1:** Data extraction table for the reviewed studies TO: transosseous technique; SA: suture anchor technique; TS: transosseous suture; IKDC: International Knee Documentation Committee score; KOOS: knee injury and osteoarthritis outcome score; VAS: visual analog scale for pain; MPFL: medial patellofemoral ligament; PT/BW: peak torque to body weight ratio; PFOA: patellofemoral osteoarthritis; ROM: range of motion

Variable	Yoon et al. (2020) [16]	Ye et al. (2020) [17]	Yanke et al. (2023) [12]	Plesser et al. (2018) [18]
Study Design	Cohort Study	Prospective Nonrandomized Controlled Trial	Retrospective Cohort Study	Pilot Study
Sample Size	46 (TO: 21, SA: 25)	65 (TS: 34, SA: 31)	68 (TO: 33, SA: 35)	17 (TS: 8, SA: 9)
Patient Demographics	TO: Mean age 24.4 ± 6.1 years; 52.4% male SA: Mean age 24.1 ± 12.1 years; 28.0% male	TS: Mean age 28.15 ± 6.01 years; 19 females, 15 males SA: Mean age 28.90 ± 5.68 years; 18 females, 13 males	TO: Mean age 54.75 ± 12.85 years; 31 males, 2 females SA: Mean age 53.98 ± 12.48 years; 32 males, 3 females	TS: Mean age 57.9 ± 12.7 years, all male SA: Mean age 62.7 ± 8.8 years, all male
Intervention Details	MPFL reconstruction with TO fixation or SA fixation	MPFL reconstruction using transosseous sutures or suture anchors	Quadriceps tendon repair using transosseous tunnels or suture anchors	Quadriceps tendon repair using transosseous sutures or suture anchors
Follow-up Duration	2 years	25-60 months	Mean of 4.81 ± 2.60 years	46 months (SA group), 29 months (TS group)
IKDC Score	TO (pre 38.5 ± 15.3, post 83.2 ± 10.8) SA (pre 35.1 ± 14.4, post 86.6 ± 10.0)	TS (pre 48.87 ± 9.10, post 89.52 ± 4.69) SA (pre 47.94 ± 7.32, post 87.68 ± 4.18)	SA: 79.6 (50.0-93.6) TO: 62.1 (44.3-65.5), p = 0.048	SA (76.0 ± 13.9) TS (85.1 ± 7.1)
Lysholm Score	TO (pre 37.4 ± 15.1, post 83.1 ± 12.6) SA (pre 45.6 ± 19.4, post 89.3 ± 10.9)	TS (94 ± 7) SA (88 ± 10)	Satisfaction: SA (10 (10-10)) vs. TO (10 (7.5-10), p = 0.04)	SA (88 ± 10); TS (94 ± 7)
Kujala Score	N/A	TS (pre 55.13 ± 8.98, post 88.90 ± 3.75) SA (pre 55.52 ± 8.75, post 89.06 ± 3.37)	N/A	N/A
Tegner Activity Score	TO (pre 2.8 ± 1.4, post 5.8 ± 1.4) SA (pre 2.5 ± 1.2, post 4.9 ± 1.2)	TS (5 (3-7)) SA (4 (3-5))	N/A	N/A
Congruence Angle	TO (pre 3.9 ± 31.9, post -3.2 ± 22.8) SA (pre -8.7 ± 22.2, post -7.6 ± 17.8)	TS (pre 21.03 ± 4.53, post -6.61 ± 5.10) SA (pre 21.74 ± 4.09, post -6.94 ± 5.82)	N/A	N/A
Patellar Tilt	TO: pre 18.4 ± 10.8, post 10.5 ± 5.4 SA: pre 14.6 ± 4.8, post 13.7 ± 2.8	TS (pre 22.35 ± 4.03, post 10.45 ± 3.10) SA (pre 22.76 ± 3.62, post 9.79 ± 3.23)	N/A	N/A
KOOS Pain	N/A	SA (97.2 (84.7-97.2)) vs. TO (73.6 (50.7-88.2), p = 0.037)	N/A	N/A
VAS for Pain	N/A	SA (5 ± 6) TS (0 ± 0)	N/A	N/A
Isokinetic Strength Testing (PT/BW)	N/A	N/A	N/A	SA (122.5 ± 44.9); TS (158.9 ± 41.9)
Complications	TO group had significantly more complications (redislocation, patellar fracture, PFOA progression)	No significant differences in outcome scores, ROM, congruence angle, patellar tilt, or redislocation rate between groups	No re-ruptures in either group. Four patients in the SA group, compared to none in the TS group, indicated mild residual pain on the VAS	Most failures were due to traumatic reinjury within the first year postoperatively
Conclusions	Both TO and SA fixation methods improved clinical outcomes. SA fixation presented comparable clinical outcomes and a lower complication rate compared with TO fixation	MPFL reconstruction using either transosseous sutures or suture anchors achieved good clinical outcomes with no significant differences between the techniques	No significant difference in failure rate between TO and SA repairs. SA repairs may result in greater subjective function and better IKDC, KOOS Pain, Quality of Life, and Sport scores	Both SA and TS repairs provided good to excellent outcomes. No significant differences in clinical outcomes were observed between the two techniques. SA repair may offer intraoperative benefits and enable earlier rehabilitation

Quality assessment of the included studies

The quality of the included studies was evaluated using the NOS, which is a tool designed to assess the risk of bias in non-randomized studies. Each study was evaluated across three broad categories: selection of participants, comparability of study groups, and outcome/exposure assessment. The assessment results for each study are summarized in Table 2, reflecting their performance in addressing potential biases.

**Table 2:** The Newcastle-Ottawa Scale (NOS) Assessments for the Studies Selection: Evaluates the representativeness of the study sample and the process of case selection (Max 4 stars). Comparability: Assesses whether the study controls for important confounding variables (Max 2 stars). Outcome/Exposure: Focuses on the methods used to assess outcomes or exposures and the adequacy of the follow-up (Max 3 stars).

Study Reference	Selection (Max 4 Stars)	Comparability (Max 2 Stars)	Outcome/Exposure (Max 3 Stars)	Total Stars (Max 9 Stars)
Yoon et al. (2020) [16]	★★★★	★☆	★★★	9.0
Ye et al. (2020) [17]	★★★★	★☆	★★★	8.5
Yanke et al. (2023) [12]	★★★★	★☆	★★★	8.5
Plesser et al. (2018) [18]	★★★★	☆☆	★★☆	6.5

Explanation of the Table

Selection: This category evaluates how well the studies selected their cohorts, including representativeness of the sample and clarity of case definitions. Comparability: This evaluates the comparability between groups in the study, particularly whether they controlled for confounding variables. Outcome/exposure: This looks at the adequacy of outcome/exposure measures, including follow-up duration and the reliability of assessments. Total stars: The overall score out of a maximum of nine stars, representing the study's quality in mitigating biases.

Key Points

Yoon et al. (2020) [16], Ye et al. (2020) [17], and Yanke et al. (2023) [12] were strong in selection and outcome/exposure categories, earning near full marks. Plesser et al. (2018) [18] had a lower score due to weaker comparability controls and fewer stars in outcome/exposure, possibly due to limited follow-up.

Stars in comparability: Some studies received fewer stars in this category due to less rigorous control of confounding variables between comparison groups

Results of meta-analysis

IKDC Score

The IKDC score was assessed across four studies included in our meta-analysis. Due to significant heterogeneity observed among the studies (I^2^ = 93%), a random-effects model was employed for the meta-analysis. The findings indicated that the SMD in postoperative IKDC scores between the transosseous and suture anchor groups was not statistically significant, with a p-value of 0.61. The overall effect size (SMD) was -0.31, with a 95% CI ranging from -1.51 to 0.89.

**Figure 2:**
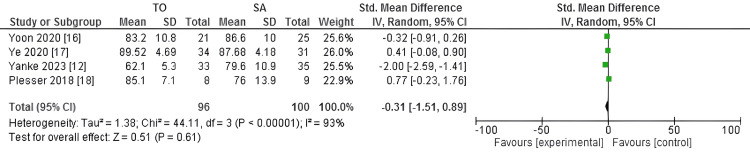
The forest plot of the comparison of IKDC scores between the transosseous (TO) and suture anchor (SA) groups IKDC: International Knee Documentation Committee; CI: confidence interval; SMD: standardized mean difference

The forest plot (Figure 2) provides a visual representation of the comparison of IKDC scores between the transosseous and suture anchor groups. It displays the CI and the SMD, helping to illustrate the distribution and relative impact of each study within the overall analysis.

Publication Bias Assessment

**Figure 3:**
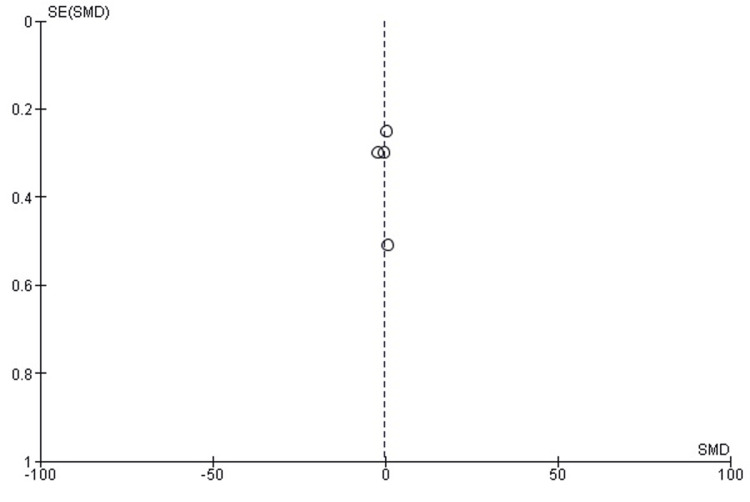
Funnel plot (IKDC score) IKDC: International Knee Documentation Committee; SMD: standard mean difference

A funnel plot analysis was conducted to evaluate the symmetry of the data, specifically for the IKDC score indicator (Figure 3). The results from Egger's test did not suggest significant publication bias, with a p-value greater than 0.05, indicating a balanced distribution of the studies. This supports the reliability of the meta-analysis findings.

The funnel plot for the IKDC score further corroborates the absence of publication bias, illustrating the spread and symmetry of the included studies around the effect size. This visual presentation reinforces that the reporting of the studies is not skewed, enhancing the confidence in the results derived from the meta-analysis.

Discussion

Understanding the biomechanics involved in patellar tendon repair is essential for optimizing knee function and supporting early mobilization. The choice between suture anchor and transosseous tunnel techniques presents a complex decision for surgeons, as each method offers distinct advantages and challenges. This systematic review and meta-analysis sought to evaluate and compare these two repair strategies in terms of biomechanical performance, functional outcomes, and patient-centered measures such as quality of life and satisfaction.

Both techniques demonstrated significant improvements in postoperative functional outcomes, with scores on the IKDC, Lysholm, Tegner, Kujala, and KOOS scales showing substantial enhancement across studies [7]. Additionally, specific patient-centered outcomes, such as pain reduction and patient satisfaction, were favorable across both methods. Sherman et al. [20] found that suture anchor and transosseous techniques effectively lowered postoperative pain levels, as reflected by reductions in VAS scores. Mehta et al. [21] supported these findings, noting that both techniques led to successful pain management and high patient satisfaction levels. The suture anchor technique, in particular, appeared to provide better patient satisfaction and quality of life outcomes, likely due to lower complication rates and faster recovery times, which are crucial for patient-centered care and long-term well-being.

The rate of complications differed between the techniques, with the suture anchor method showing a lower incidence compared to TO. O'Donnell et al. [22] reported higher complication rates in patients treated with transosseous repairs, including issues such as redislocation, patellar fractures, and progression of patellofemoral osteoarthritis. In contrast, the suture anchor technique demonstrated a lower incidence of these complications, suggesting a potentially safer profile for patients. This observation aligns with Massey et al. [10], who also highlighted lower complication rates in the suture anchor group. Lower complication rates are directly associated with better patient outcomes, as fewer complications often lead to faster recovery, less postoperative pain, and a reduced need for reoperation. These factors significantly affect quality of life and patient satisfaction.

Regarding follow-up duration, this review found some variability in short-term and long-term outcomes. The studies showed that the suture anchor technique often led to quicker improvements in pain and function, making it potentially more beneficial in the short term. However, differences between the suture anchor and transosseous techniques in long-term outcomes were less clear, and both methods appeared to support satisfactory knee function over extended follow-up periods. Ettinger et al. [19] observed that the biomechanical stability provided by suture anchors could accelerate early recovery, but no significant differences were reported in functional scores after one to two years of follow-up. Long-term studies with consistent follow-up durations would be valuable to clarify whether either technique offers superior durability in functional outcomes or quality-of-life metrics.

Postoperative rehabilitation is a critical factor influencing recovery, yet this review identified limited information on the specific protocols followed in each study. Variations in rehabilitation intensity, timing, and duration likely contributed to some of the heterogeneity observed in functional outcomes. Standardizing rehabilitation protocols across studies could allow for a clearer comparison of the techniques' intrinsic benefits, potentially enhancing recovery times and minimizing complications. Given the importance of physiotherapy and structured rehabilitation programs in achieving optimal tendon healing and functional recovery, future research should incorporate detailed rehabilitation protocols to improve the applicability of findings and guide clinical practice.

Finally, while our meta-analysis identified notable improvements with both techniques, the effect sizes observed were moderate, and clinical significance may vary based on individual patient factors. The pooled SMD in IKDC scores, for instance, favored the suture anchor technique slightly but did not reach statistical significance. This suggests that although both techniques are effective, the choice of method may need to be personalized based on factors such as patient age, activity level, and specific biomechanical needs. The high degree of heterogeneity across studies (I^2^ = 93%) also suggests that differences in patient populations, surgical approaches, and outcome measures may affect the generalizability of the results [23].

In summary, both suture anchor and transosseous techniques produce favorable biomechanical and functional outcomes in patellar tendon repair, with the suture anchor method showing potential advantages in early rehabilitation and complication rates. Incorporating standardized rehabilitation protocols and expanding on long-term follow-up studies would provide further insights into the effectiveness and patient-centered impact of each technique. These findings underscore the importance of a holistic approach in surgical planning, focusing not only on biomechanical success but also on quality of life, pain management, and patient satisfaction [23].

Limitations

This meta-analysis includes a limited number of studies with notable heterogeneity, which may impact the generalizability of the results. Differences in study design, patient demographics, and follow-up durations likely contributed to this variability. However, despite these limitations, our findings provide valuable insights into the comparative biomechanics and functional outcomes of suture anchor versus transosseous tunnel techniques in patellar tendon repair. Additionally, potential biases in the original studies may influence the conclusions drawn; therefore, careful interpretation is advised. We recommend that future investigations apply more standardized methodologies and larger, homogeneous sample populations to build upon these results and establish robust clinical guidelines.

## Conclusions

Both the suture anchor and transosseous tunnel techniques demonstrate effectiveness in patellar tendon repair, contributing to meaningful functional improvements and stability. Our meta-analysis found no significant difference in IKDC scores between the methods, although suture anchor may offer practical advantages, including faster rehabilitation and potentially fewer complications. Clinically, these differences suggest that the suture anchor technique could be particularly beneficial for patients requiring quicker recovery times or those with a higher risk of complications. In terms of patient-centered outcomes, suture anchor's lower complication rates and faster rehabilitation timelines may translate to an improved quality of life and higher patient satisfaction, particularly relevant for patients engaged in active or physically demanding lifestyles. This indicates that while both techniques are viable, the suture anchor method might be the preferred choice when minimizing downtime is critical to patient recovery goals.

For clinical practice, we recommend selecting the suture anchor over the transosseous tunnel technique in cases where early mobilization and reduced complication risk are priorities, while the transosseous tunnel may remain suitable in contexts where cost or equipment limitations are a factor. Further research should explore specific patient profiles that might benefit from one technique over the other, as well as the impact of comprehensive, standardized rehabilitation protocols on long-term outcomes. Standardizing these protocols across patient populations could help optimize recovery timelines and patient satisfaction, thus enhancing the broader clinical impact of tendon repair techniques.
